# Assessment of Cerebral and Cerebellar White Matter Microstructure in Spinocerebellar Ataxias 1, 2, 3, and 6 Using Diffusion MRI

**DOI:** 10.3389/fneur.2020.00411

**Published:** 2020-06-04

**Authors:** Young Woo Park, James M. Joers, Bin Guo, Diane Hutter, Khalaf Bushara, Isaac M. Adanyeguh, Lynn E. Eberly, Gülin Öz, Christophe Lenglet

**Affiliations:** ^1^Department of Radiology, Center for Magnetic Resonance Research, University of Minnesota Medical School, Minneapolis, MN, United States; ^2^Division of Biostatistics, School of Public Health, University of Minnesota, Minneapolis, MN, United States; ^3^Department of Neurology, University of Minnesota Medical School, Minneapolis, MN, United States

**Keywords:** SCA1, SCA2, SCA3, SCA6, diffusion MRI, *Spinocerebeflar ataxias*

## Abstract

Development of imaging biomarkers for rare neurodegenerative diseases such as spinocerebellar ataxia (SCA) is important to non-invasively track progression of disease pathology and monitor response to interventions. Diffusion MRI (dMRI) has been shown to identify cross-sectional degeneration of white matter (WM) microstructure and connectivity between healthy controls and patients with SCAs, using various analysis methods. In this paper, we present dMRI data in SCAs type 1, 2, 3, and 6 and matched controls, including longitudinal acquisitions at 12–24-month intervals in a subset of the cohort, with up to 5 visits. The SCA1 cohort also contained 3 premanifest patients at baseline, with 2 showing ataxia symptoms at the time of the follow-up scans. We focused on two aspects: first, multimodal evaluation of the dMRI data in a cross-sectional approach, and second, longitudinal trends in dMRI data in SCAs. Three different pipelines were used to perform cross-sectional analyses in WM: region of interest (ROI), tract-based spatial statistics (TBSS), and fixel-based analysis (FBA). We further analyzed longitudinal changes in dMRI metrics throughout the brain using ROI-based analysis. Both ROI and TBSS analyses identified higher mean (MD), axial (AD), and radial (RD) diffusivity and lower fractional anisotropy (FA) in the cerebellum for all SCAs compared to controls, as well as some cerebral alterations in SCA1, 2, and 3. FBA showed lower fiber density (FD) and fiber crossing (FC) regions similar to those identified by ROI and TBSS analyses. FBA also highlighted corticospinal tract (CST) abnormalities, which was not detected by the other two pipelines. Longitudinal ROI-based analysis showed significant increase in AD in the middle cerebellar peduncle (MCP) for patients with SCA1, suggesting that the MCP may be a good candidate region to monitor disease progression. The patient who remained symptom-free throughout the study displayed no microstructural abnormalities. On the other hand, the two patients who were at the premanifest stage at baseline, and showed ataxia symptoms in their follow-up visits, displayed AD values in the MCP that were already in the range of symptomatic patients with SCA1 at their baseline visit, demonstrating that microstructural abnormalities are detectable prior to the onset of ataxia.

## Introduction

Spinocerebellar ataxias (SCAs) are rare neurodegenerative diseases characterized by progressive gait and balance problems. A systematic review of SCAs showed a global prevalence of the disease at 3 out of 100,000 (1, 2), with the most common SCAs being types 1 (SCA1), 2 (SCA2), 3 (SCA3), and 6 (SCA6) (3). Together, these four SCAs, which are polyglutamine (polyQ) disorders caused by expansion of CAG triplet repeats, account for more than half of all patients with SCAs. Analysis of clinical features as well as disease pathology has revealed differences among the subtypes (4, 5). SCA1 is the first genetically identified (6) SCA with the fastest progression among SCA1, 2, 3, and 6 and characterized by atrophy mainly in the pons and cerebellum (7). SCA2 shows the most severe and widespread atrophy among common SCAs in both cerebrum and cerebellum (8). SCA3 is the most common SCA and shows atrophy throughout the cerebrum and cerebellum (9). SCA6 shows almost pure cerebellar atrophy and has later onset and slower progression compared to other types (10). Post-mortem neuropathology studies revealed loss of Purkinje neurons in gray matter as well as demyelination, loss of fibers, and axonal swellings in white matter (WM) in SCAs (5).

Development of imaging biomarkers for SCAs is important to non-invasively track progression of disease pathology and monitor response to interventions. Hence, previous studies utilized MR imaging techniques such as volumetry (11–14) and spectroscopy (15–17) to show macrostructural and neurochemical differences between participants with different SCA types and healthy controls. In addition, there are efforts to understand the progression of disease by performing cross-sectional analysis in groups at different stages of the disease (18) or longitudinal analysis with follow-up measurements of the same subjects (14, 19). Diffusion MRI (dMRI) allows visualization and characterization of the WM microstructure and axonal pathways and can be used to detect alterations in WM integrity and connectivity in patients with SCAs. Several cross-sectional dMRI studies in SCAs using 3T MRI have been reported and showed significant differences in the brainstem and cerebellum between healthy controls and patients (20–25) using either region of interest (ROI) (26, 27), tract-based spatial statistics (TBSS) (28), or fixel-based analysis (FBA) (29) methods. In addition, longitudinal studies on SCA2 cohorts reported widespread changes in WM within both the cerebellum and cerebrum using TBSS at 1.5T MRI (30, 31). To date, there is no longitudinal study investigating longitudinal degeneration patterns in SCA 1, 2, 3, and 6 with a dMRI protocol at 3T.

In this study, we have collected longitudinal dMRI datasets from patients with SCA1, 2, 3, and 6, using high angular resolution diffusion imaging (HARDI) at 3T, and performed a cross-sectional and longitudinal evaluation of white matter (WM) alterations. Since dMRI data can be processed in a variety of ways prior to statistical analysis, we hypothesized that different analysis pipelines may yield unique and complementary outcomes. Aggregation of such results could help better identify WM microstructural abnormalities in SCA1, 2, 3, and 6. In addition, we investigated longitudinal alterations by measuring the change in dMRI metrics in various WM ROIs in the same cohorts. Finally, we report associations between dMRI metrics and clinical scores.

## Materials and Methods

### Participants and Study Design

Thirty-five genetically confirmed patients with SCA1, 2, 3, and 6 (*N* = 11, 9, 7, and 8) and 9 healthy controls were scanned for this study. A subset of the cohort (*N* = 9, 5, 4, and 5 for SCA1, 2, 3, and 6, respectively) underwent the same protocol up to four more times, with 12–24 month intervals. The study targeted patients in the early disease stage and therefore primarily focused on patients with a scale for the assessment and rating of ataxia (SARA) (32) score of 15 or lower at baseline (0 = no ataxia / 40 = most severe ataxia). The study included three premanifest patients with SCA1, with SARA≤2.5 at baseline (33). Using a statistical model based on genotype (34), estimated time to ataxia onset was computed for the premanifest patients at baseline. The control group mean was matched to each SCA group with respect to age, sex, and BMI (Table 1). Healthy controls were scanned only once. Body mass index (BMI) and quality of life score using the activities of daily living (ADL) portion of the Friedreich's Ataxia Rating Scale (35, 36) were collected prior to the MRI scan. Disease duration and CAG repeat lengths of each patient were also recorded.

**Table 1:** Participant demographics and clinical characteristics at baseline.

	**Demographics at baseline**								
	**N (Males/Females)**	**Age at scan (yr)**	**Age at onset (yr)**	**Age of diagnosis (yr)**	**Disease duration (yr)**	**CAG repeat length**	**SARA score**	**ADL score**	**BMI (kg/m^**2**^)**
SCA1	11 (4/7)	49 ± 12	40 ± 11	40 ± 14	10 ± 6	45 ± 4	7.1 ± 5.7	6.8 ± 5.3	26.6 ± 3.6
SCA2	9 (5/4)	43 ± 15	34 ± 10	38 ± 12	12 ± 8	39 ± 3	10.0 ± 4.1	6.1 ± 4.2	25.0 ± 4.0
SCA3	7 (4/3)	49 ± 6	41 ± 7	44 ± 7	8 ± 5	71 ± 2	7.4 ± 2.5	6.4 ± 4.1	24.9 ± 2.3
SCA6	8 (2/6)	66 ± 8	53 ± 14	58 ± 12	13 ± 7	22 ± 2	13.6 ± 8.9	10.6 ± 7.1	25.0 ± 2.7
Control	9 (4/5)	54 ± 16	–	–	–	–	0.0 ± 0.0	0.0 ± 0.0	24.2 ± 6.0

*Disease duration was computed as the difference between reported age at onset and age at scan. Age at onset is the self-reported age when the patient had ataxia symptoms, and age at diagnosis was when the presence of SCA mutation was genetically confirmed. Three patients (2 SCA1 and 1 SCA2) were diagnosed with genetic testing before they experienced ataxia symptoms, and their age at onset and age of diagnosis are excluded in this table. Scale for the assessment and rating of ataxia (SARA), quality of life score using the activities of daily living (ADL) portion of the Friedreich's Ataxia Rating Scale, and body mass index (BMI) scores were measured at the time of each scan. Reported values are mean ± SD*.

During the study, the MRI scanner underwent an upgrade from the Trio to Prisma Fit platform, which only affected dMRI datasets for patients with SCA1. We recruited 5 new healthy participants (2 females, aged 29 ± 5) and acquired dMRI datasets using the same protocol, in order to investigate whether the upgrade had any significant effect on extracted diffusion metrics. Each subject was scanned before and after the upgrade, all within a 2-week period.

The study protocol was approved by the University of Minnesota Institutional Review Board, and all participants provided signed an informed consent.

### Diffusion MRI Acquisition and Pre-processing

All data from healthy controls and patients with SCA2, 3, and 6 were acquired using a Siemens (Erlangen, Germany) 3T Trio MRI scanner. Due to an upgrade of the Trio scanner to the Prisma Fit platform during the course of the study, data from 7 subjects with SCA1 were acquired on the Trio platform, data from 2 subjects with SCA1 were acquired on the Prisma platform, and 2 subjects with SCA1 started the study on Trio and then switched to Prisma. dMRI data were collected using a HARDI protocol with 1.8 × 1.8 × 1.8-mm^3^ spatial resolution, matrix size 106 × 106, 90 slices, TR/TE = 4253/90.6 ms, no in-plane acceleration, *b* = 1,500 s/mm^2^, and 128 gradients uniformly distributed with 15 additional *b* = 0 s/mm^2^ volumes. Two sets of data were acquired in anterior–posterior (AP) and posterior–anterior (PA) phase-encoding directions, and the pair was used to correct for geometric distortions using the diffusion processing pipeline in FSL (FDT) (37). The acquisition protocol was exactly matched on the Prisma platform, with the exception of a slightly shorter echo spacing (0.66 vs. 0.69 ms on Trio). The standard Siemens 32 channel head matrix receive coil was used on both Trio and Prisma platforms for the main study on the patients with SCAs. For the scanner upgrade validations with 5 healthy volunteers, the 32-channel head coil was used to acquire dMRI data before the upgrade, and the 64-channel head/neck coil was used after the upgrade.

### Analysis of dMRI Data

Based on previously reported dMRI studies in SCAs, we compared three different dMRI processing pipelines. Each SCA subtype was analyzed separately due to differences in the pathology between subtypes. First, we considered ROI-based analyses focused on dMRI metrics extracted from WM regions broadly related to SCA (20, 22) and often involved in other neurodegenerative disorders. Voxel-based TBSS analysis was also employed in many dMRI studies (21, 23–25, 30). Finally, fixel-based analysis (FBA) (25) was recently used to identify differences in estimated WM fiber density and crossing by utilizing high-order diffusion models. The pre-processed dMRI data were analyzed with these three methods, and their results were compared.

#### ROI-Based Analysis

**Figure 1:**
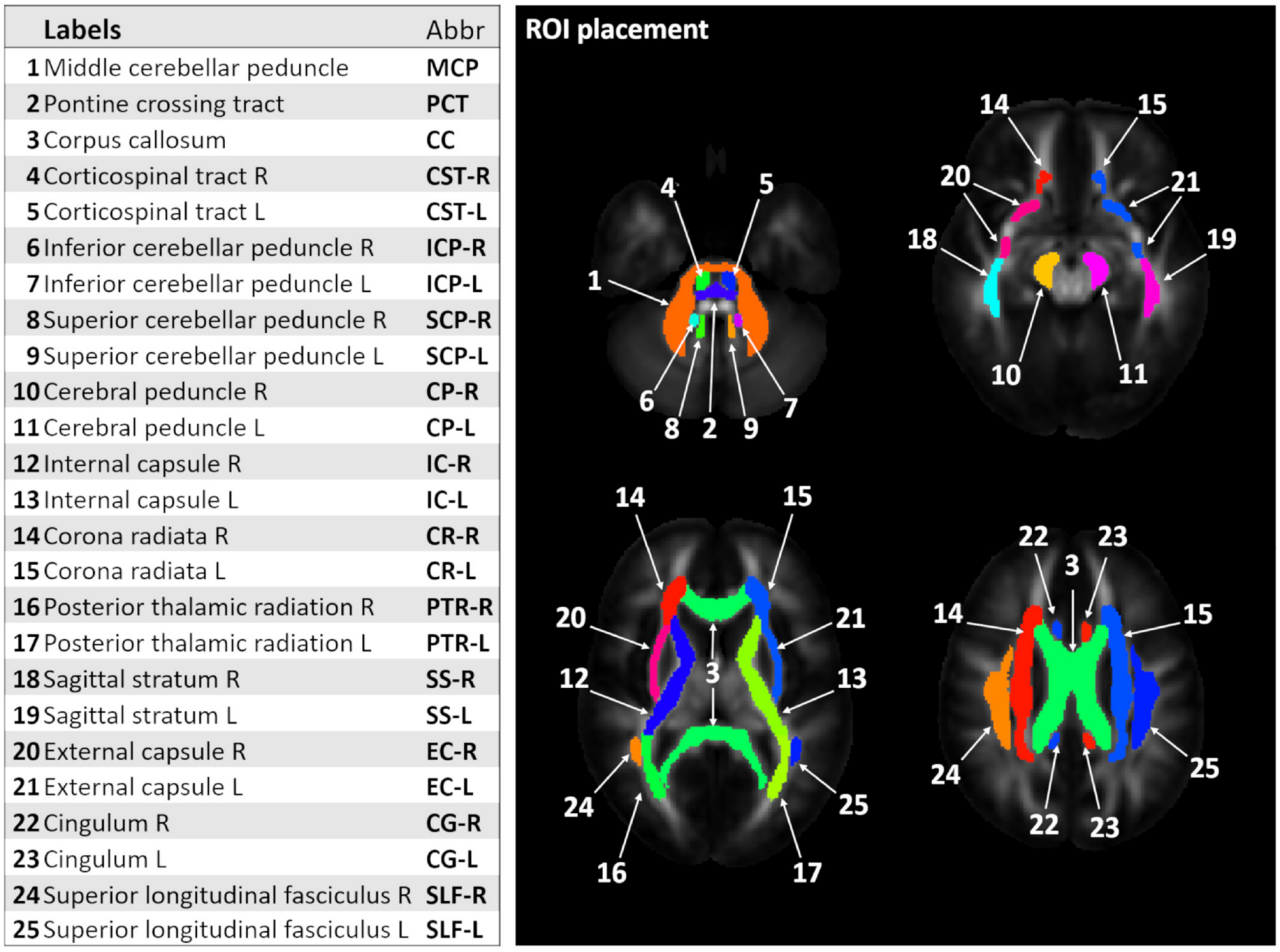
Labels and corresponding abbreviations for region-of-interest (ROI) analysis. The figure shows 25 white matter (WM) ROI labels identified using the Johns Hopkins (JHU) WM template and atlas (13).

From the pre-processed data, fractional anisotropy (FA) and mean (MD), axial (AD), and radial diffusivity (RD) maps were generated in each subject's native space. Since many pathological studies in SCAs report alterations in both cerebellum and cerebrum, we used a whole brain WM atlas to identify WM ROIs. The Johns Hopkins (JHU) WM FA template and atlas (38) were used to automatically locate the ROIs in each subject's native space. We focused on 25 WM tracts, as shown on Figure 1. ANTs were used to locate the WM ROI for each participant by non-linearly registering the JHU WM template to their respective FA map.

#### Tract-Based Spatial Statistics (TBSS) Analysis

TBSS (28) is a widely used voxel-based analysis method for dMRI data, which can identify changes in WM integrity. However, it requires all datasets to be aligned in a common space prior to TBSS and subsequent statistical analysis. We utilized the tensor-based DTI-TK (39, 40) spatial normalization technique instead of FSL's FA-based normalization for improved alignment to the template (41).

#### Fixel-Based Analysis (FBA)

FBA attempts to improve the interpretability of voxel-averaged measures such as FA by computing fiber orientation distributions (FOD) that allow the estimation of fiber density (FD) and fiber-bundle cross-section (FC) metrics. WM FOD metrics were estimated using MRtrix3 (29) with multi-tissue constrained spherical deconvolution (CSD), accounting for WM and gray matter (GM) compartments (42). Preprocessing steps such as bias field correction and inter-subject spatial normalization were also performed using the tools within the MRtrix3 package. The FBA pipeline yields FD, FC, and the combined measure of fiber density and bundle cross-section (FDC), to characterize the WM microstructure.

### Statistical Analysis

#### Effect of Scanner Upgrade

dMRI datasets of the five healthy subjects measured before and after upgrade were compared to find any significant effect of scanner upgrade on extracted dMRI metrics. ROI-based analysis was performed with six ROIs on JHU WM Atlas: middle cerebellar peduncle (MCP); corpus callosum (splenium); posterior corona radiata (left/right); and superior longitudinal fasciculus (SLF, left/right). The analysis compared common DTI metrics such as FA and MD values. A paired *t*-test on each subject and ROI with adjustments for multiple dMRI metrics was performed with *p* < 0.05 as the threshold for significance.

#### Cross-Sectional Analysis

For this analysis, data from healthy controls (*n* = 9) were compared against the baseline data of each SCA cohort. For ROI analysis, linear models were fitted using the R software with age and sex as covariates, to compare participants with SCAs to controls for FA, MD, AD, and RD values. Our analysis of dMRI metrics assumed FA, MD, AD, and RD to be independent from each other, as widely done in analysis pipelines such as TBSS. Stepdown Bonferroni type I error correction for multiple testing (25 ROIs) was performed. For TBSS and FBA, generalized linear model (GLM) analysis between groups of patients and controls was performed with *p* < 0.05 as the significant threshold after family-wise error correction. For TBSS, statistical analysis was performed with FSL's *randomize* with 5000 iterations and threshold-free cluster enhancement. For FBA, statistical analysis was performed with *fixelcfestats* tool with 5000 permutations.

#### Longitudinal Analysis

A subset of each SCA cohort returned for follow-up scans (*N* = 9, 5, 4 and 5, for SCA1, 2, 3, and 6), allowing for a longitudinal analysis to investigate changes in WM microstructure. For each ROI and each SCA group, linear regression models were fit to estimate slopes (temporal changes in days) of dMRI metrics over the duration of their research visits for each subject. One-sample *t*-tests were used to test if the slopes were significantly different from zero, that is, any significant temporal changes present. Holm's correction method was applied within SCA groups to the *p*-values for the multiple testing across ROIs. We chose to perform a ROI-based analysis to maximize statistical power despite the relatively small sample size. Moreover, longitudinal analysis with irregular time intervals is not feasible in FSL's *randomize*.

#### Associations Between dMRI Metrics and Clinical Scores

We sought to relate dMRI metrics to clinical status of patients using two different analyses. First, linear regression models were fitted for each ROI and each SCA group to test the association between the dMRI metrics (FA/MD/AD/RD) from baseline scans and clinical scores (SARA, ADL, and disease duration) with age as a covariate. This enabled us to identify the regional microstructural abnormalities that are associated with the clinical presentation. Second, linear regression models were applied to test the association between the slope of changes in clinical scores (SARA and ADL) and slope of changes in dMRI metrics. Within each SCA group, Holm's correction method was applied to the p-values for the multiple testing across ROIs.

## Results

### Demographics and Clinical Characteristics of Cohort and Longitudinal Schedule

Age, sex, and BMI were mean-matched (*p* > 0.05) between each SCA group and healthy controls (Table 1). Thirty-one out of 35 patients had a SARA score equal to or <15 at baseline, resulting in mean SARA scores of all SCA groups lower than 14 (Table 1). One patient with SCA1 (SARA = 15.75), 1 patient with SCA2 (SARA = 18), and 2 patients with SCA6 (SARA = 22 and 26) had SARA scores higher than 15. Two of three premanifest patients (SARA ≤ 2.5) at baseline started to show ataxia symptoms during follow-up visits, while one subject remained symptom-free (SARA = 0) for the three follow-up scans. Based on the genotype-based prediction model (34), the two premanifest patients that showed ataxia symptoms at their follow-up scans each had 4 years until predicted onset of ataxia at baseline, while the premanifest patient who remained symptom-free had estimated time to ataxia onset of 10 years at baseline.

In the SCA1 group, 9 out of 11 participants returned for a second scan at 625 ± 165 days after baseline; 3 participants returned for a third scan at 1334 ± 219 days; 2 participants returned for a fourth scan at 2015 ± 69 days; and 1 participant returned for a fifth scan at 2659 days. In the SCA2 group, 5 out of 9 participants returned for a second scan at 402 ± 45 days after baseline and 1 participant returned for a third scan at 673 days. Four out of 7 patients with SCA3 returned for a second scan (return interval of 433 ± 53 days), and 5 out of 8 patients with SCA6 returned for a second scan (return interval of 437 ± 84 days).

### Effect of Scanner Upgrade

Comparison of dMRI data acquired before and after the upgrade to Prisma platform showed no significant differences in DTI metrics from six different regions (*p* > 0.05, paired 2-tailed *t*-test). Mean differences ranged from 3.1 × 10^−4^ to 2.2 × 10^−2^ for FA and 0 to 3.0 × 10^−5^ for MD, in the MCP, corpus callosum, left and right SLF, and posterior corona radiata (Supplementary Figure 1). Therefore, data obtained on the Trio and Prisma platforms were pooled in subsequent analyses.

### Cross-Sectional Analysis

#### ROI-Based Analysis

**Figure 2:**
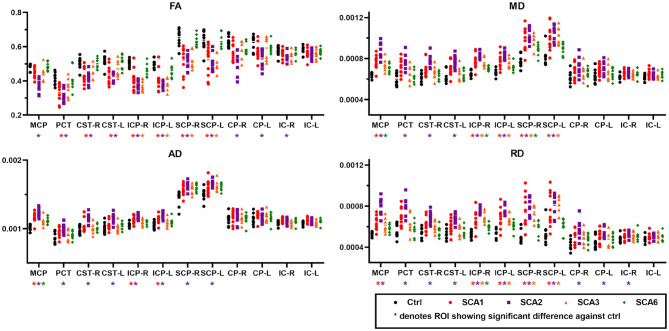
Diffusion MRI metrics from different regions-of-interest (ROI). Cross-sectional ROI analysis highlights significant differences for fractional anisotropy (FA, upper left), mean diffusivity (MD, upper right), axial diffusivity (AD, lower left), and radial diffusivity (RD, lower right) between SCAs and control groups. Abbreviations shown in the figure are as follows. MCP, middle cerebellar peduncle; PCT, pontine crossing tract; CST-R and CST-L, right and left corticospinal tract; ICP-R and ICP-L, right and left inferior cerebellar peduncle; SCP-R and SCP-L, right and left superior cerebellar peduncle; CP-R and CP-L, right and left cerebral peduncle; IC-R and IC-L, right and left internal capsule.

The ROI-based analysis of cross-sectional data for patients with SCA1 and healthy controls (Figure 2) identified significant differences (type I error corrected) bilaterally in the corticospinal tracts (*p* < 0.02 for FA), MCP (*p* < 0.01 for MD, AD, and RD), inferior cerebellar peduncle (ICP, *p* < 0.01 for FA, MD, and RD), superior cerebellar peduncle (SCP, *p* < 0.03 for FA, MD, and RD), and pontine crossing tracts (*p* < 0.04 for FA, MD, and RD).

The SCA2 group showed the greatest number of ROIs with significant differences in bilateral cerebral peduncles (*p* < 0.03 for FA and RD), in addition to FA, MD, AD, and RD in the regions that also showed differences in SCA1 such as MCP (*p* < 0.001), bilateral ICP (*p* < 0.002), bilateral SCP (*p* < 0.02), and pontine crossing tracts (*p* < 0.03).

In SCA3, we found localized differences in the bilateral ICP (*p* < 0.03) and bilateral SCP (*p* < 0.01) for FA, MD, and RD, while SCA6 only showed differences in the MCP (*p* < 0.04 for AD and MD), right ICP (*p* < 0.02 for MD and RD), and right SCP (*p* < 0.03 for MD). Limited findings in SCA6 are in part due to the fact that the ROIs used in this study do not include peripheral WM tracts or cerebellar cortex areas (See complete statistics in Supplementary Tables).

#### TBSS Analysis

**Figure 3:**
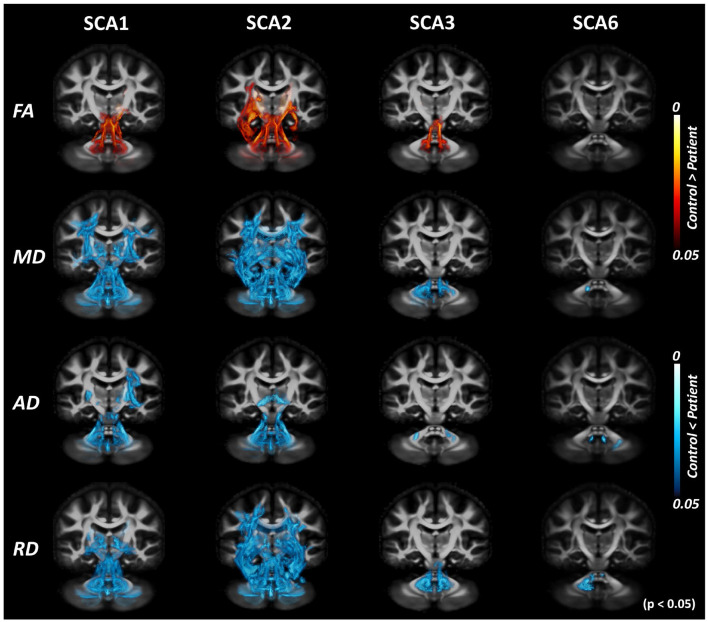
Tract-based spatial statistics (TBSS) analysis with DTI-TK spatial normalization. Volume rendering of TBSS analysis results using *ParaView* identifies voxels with significant differences between patients with SCAs and healthy controls for SCA1, 2, 3, and 6. Red regions indicate the white matter locations with significantly lower values in patients, while blue regions indicate the white matter locations with significantly higher values in patients. This illustrates consistent trends of decreased fractional anisotropy (FA) and increased diffusivity (MD/AD/RD) throughout the brain across different SCAs.

Cross-sectional TBSS analysis (Figure 3) identified the following significant (*p* < 0.05) group differences: For SCA1, bilateral differences vs. controls were seen for all metrics in the corticospinal tracts, MCP, ICP, SCP, and pontine crossing tracts. For cerebral regions, such as cerebral peduncles, internal capsules, and corona radiata, FA showed differences only in the right hemisphere, although diffusivity metrics showed bilateral differences. Only MD and AD were found to be significantly different from controls in the external capsules and superior longitudinal fasciculus, with a bilateral trend for MD and right hemisphere only for AD. The SCA2 group had the most significant and widespread differences from healthy controls, including the thalamic radiations (left only for FA, bilateral for MD and RD) and sagittal striatum (left only for MD and RD), in addition to all other regions identified in SCA1. The SCA3 group had more localized differences in the MCP (bilateral), ICP (bilateral for FA, MD, and RD), SCP (bilateral for FA, MD, and RD), and left internal capsule. The SCA6 group only showed differences in the MCP (AD and RD), ICP (left for MD), and SCP (bilateral for AD).

**Figure 4:**
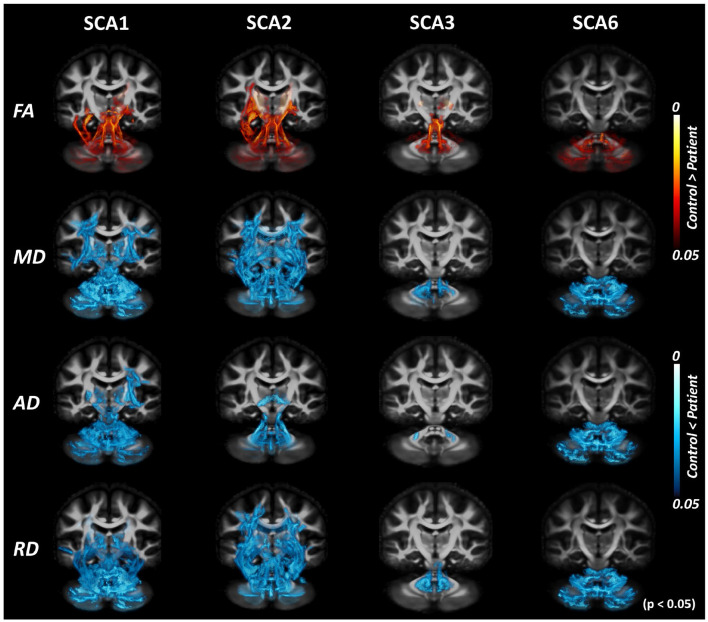
Tract-based spatial statistics (TBSS) analysis with DTI-TK spatial normalization and FA threshold of 0.1. TBSS analysis with lowered FA threshold (from 0.2 to 0.1) shows more areas of difference between patients with SCAs and healthy controls. The effect of the choice of threshold is most prominent for SCA6.

The TBSS pipeline performs intensity thresholding based on values of diffusion metrics prior to statistical analysis. The default threshold for FA is 0.2; hence, voxels with values lower than 0.2 are discarded from subsequent analyses. Figure 3 shows the result of the cross-sectional TBSS analysis using the suggested threshold of 0.2. However, with this default threshold, peripheral WM tracts in the cerebellum and cerebrum were not included. When the intensity threshold was lowered to 0.1, more areas in the cerebellum were found to show trends between SCA vs. control groups (Figure 4), which was most apparent in SCA6.

#### Fixel-Based Analysis

**Figure 5:**
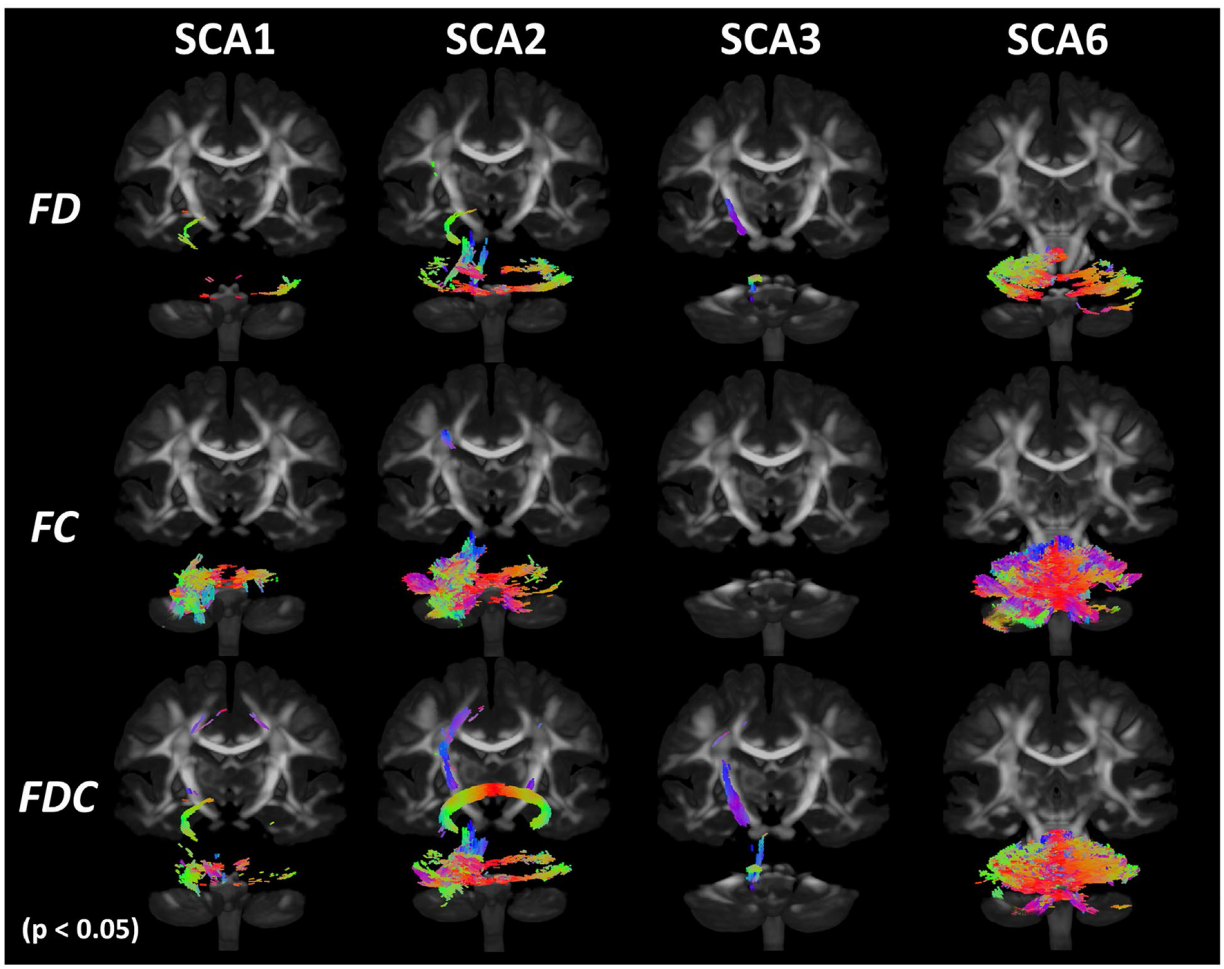
Results of fixel-based analysis (FBA). FBA analysis shows significant (*p* < 0.05 with family-wise error correction) cross-sectional differences between patients with SCAs and healthy controls for SCA1, 2, 3, and 6 in fiber density (FD), fiber crossing (FC), and FDC metrics. Colors indicate direction of the white matter fibers with blue indicating superior–inferior direction; red indicating left–right direction; and green indicating anterior–posterior direction.

Cross-sectional FBA (Figure 5 and Table 2) identified significant (*p* < 0.05) differences between the SCA1 cohort and healthy controls in the MCP, corpus callosum, and internal capsule. Consistent with ROI-based and TBSS analyses, the SCA2 group showed the most significant and widespread differences from controls among SCA1, 2, 3, and 6, including SCP, cerebral peduncle and corona radiata, superior longitudinal fasciculus, and corticospinal tract in addition to all regions identified in SCA1. The SCA3 group showed differences in the MCP, ICP, and SCP; cerebral peduncle; internal capsule; and corona radiata, while the SCA6 group showed differences in MCP and ICP. Unlike TBSS, FBA does not appear to be as dependent on the choice of thresholds (e.g., FA) and identified more widespread alterations in WM adjacent to the cerebellar cortex by better capturing crossing fiber configurations.

**Table 2:** Neuroanatomical regions showing significantly lower FD, FC, and FDC metrics in SCA1, 2, 3, and 6 cohorts than healthy controls.

**Regions**	**FD**		**FC**		**FDC**	
	**RL, AP, FH**	***p***	**RL, AP, FH**	***p***	**RL, AP, FH**	***p***
**CONTROL VS. SCA1**						
Middle cerebellar peduncle	–		−13, −43, −32	≤ 0.001	−23, −52, −32	0.006
Corpus callosum	−17, −51, 12	0.038	–		−19, −54, 14	0.018
Inferior cerebellar peduncle L	–		−11, −41, −36	0.001	–	
Internal capsule L	–		–		−20, −15, 0	0.043
Posterior thalamic radiation L	−26, −68, 15	0.049	–		−24, −62, 15	0.028
Others (unclassified)	37, −57, −29	0.010	–		−6, −64, −22	0.005
**CONTROL VS. SCA2**						
Middle cerebellar peduncle	−14, −33, −26	≤ 0.001	−21, −58, −36	≤ 0.001	−15, −37, −29	≤ 0.001
Corpus callosum	−22, −61, 13	0.023	–		−22, −57, 12	0.002
Corticospinal tract L	−10, −29, −22	0.032	−10, −29, −22	0.001	−10, −29, −22	0.002
Inferior cerebellar peduncle L	−8, −44, −37	0.005	−9, −40, −42	≤ 0.001	−10, −41, −40	≤ 0.001
Superior cerebellar peduncle L	−7, −43, −30	0.014	−7, −41, −30	0.018	−7, −43, −30	0.016
Cerebral peduncle R	–		–		21, −18, −5	0.022
Cerebral peduncle L	−10, −29, −21	0.002	−10, −29, −21	≤ 0.001	−10, −29, −21	≤ 0.001
Internal capsule R	–		–		21, −17, −4	0.022
Internal capsule L	–		–		−17, −15, 2	0.016
Corona radiata L	−30, −19, 26	0.04	−23, −20, 39	0.016	−21, −14, 22	0.03
Posterior thalamic radiation R	–		–		29, −61, 13	0.004
Posterior thalamic radiation L	−24, −65, 15	0.026	–		−25, −64, 14	0.002
Superior longitudinal fasciculus L	−31, 2, 22	0.019	−26, −21, 40	0.033	−26, −21, 40	0.043
Others (unclassified)	−14, −33, −25	≤ 0.001	−22, −65, −46	≤ 0.001	−29, −57, −37	≤ 0.001
**CONTROL VS. SCA3**						
Middle cerebellar peduncle	–		–		−10, −39, −41	0.021
Inferior cerebellar peduncle L	−7, −52, −24	0.001	–		−8, −50, −26	0.001
Superior cerebellar peduncle L	–		–		−5, −42, −30	0.026
Cerebral peduncle L	−14, −19, −15	0.023	–		−16, −17, −7	0.004
Internal capsule L	−19, −15, −3	0.013	–		−17, −16, −4	0.004
Corona radiata L	–		–		−20, −14, 19	0.031
Superior longitudinal fasciculus L	–		–		−28, −20, 37	0.027
Others (unclassified)	−9, −44, −46	0.003	–		−9, −52, −26	0.002
**CONTROL VS. SCA6**						
Middle cerebellar peduncle	–		−25, −51, −32	≤ 0.001	−23, −51, −30	≤ 0.001
Inferior cerebellar peduncle R	–		13, −49, −23	0.001	12, −50, −23	0.009
Inferior cerebellar peduncle L	–		−11, −48, −24	0.001	−10, −51, −22	0.015
Others (unclassified)	−32, −61, −25	≤ 0.001	5, −66, −25	≤ 0.001	2, −67, −25	≤ 0.001

*White matter areas with significant differences are indicated with their corresponding location (MNI coordinates) and p-value of local maxima*.

### Longitudinal ROI Analysis

**Figure 6:**
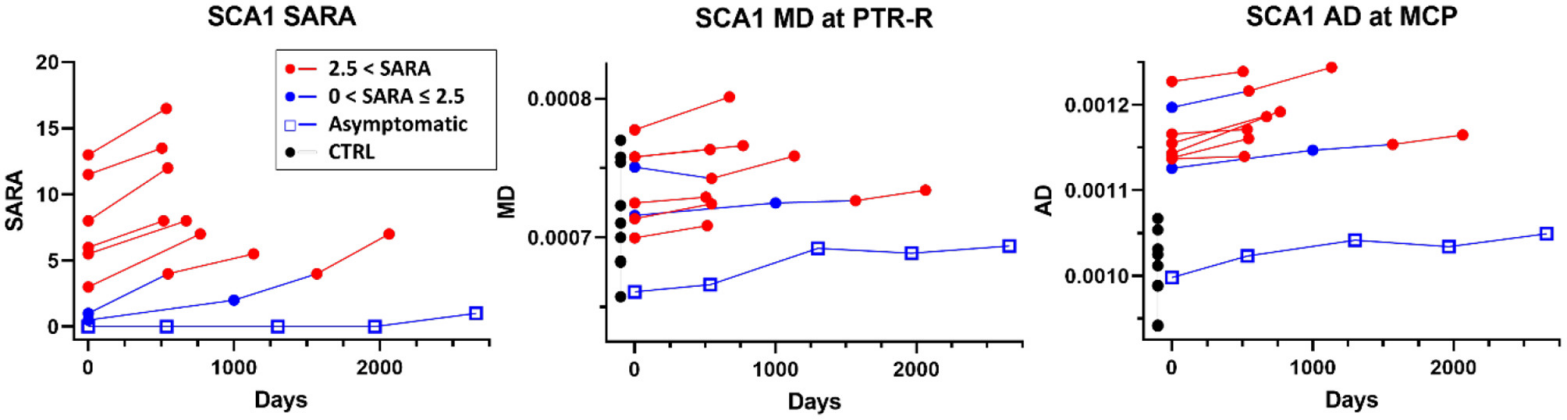
Results of longitudinal ROI-based analysis. Significant changes (slope different than 0) were detected for mean diffusivity (MD) in the right posterior thalamic radiation (PTR-R) and for axial diffusivity (AD) in the middle cerebellar peduncle (MCP) in SCA1. The data acquired from participants at pre-manifest stages are indicated in blue, with a single premanifest patient, who remained at Scale for the Assessment and Rating of Ataxia (SARA) <2 throughout the study indicated with blue open squares. Black circles indicate values from healthy controls in the same regions. SARA scores of the same patients are shown on the left.

Significant longitudinal changes (Figure 6) were identified with increasing MD in the right posterior thalamic radiations (PTR-R, *p* = 0.027) and increasing AD in the MCP (*p* = 0.043) in patients with SCA1. AD of the MCP also showed a clear separation between patients with SCA1 and healthy controls and was already abnormal (high) in two subjects who were at the premanifest stage at enrollment. The complete statistics including mean slopes of DTI metrics, standard deviations of slopes, and type I error corrected p-values were summarized in Supplementary Tables.

### Associations Between dMRI Metrics and Clinical Scores

Association between the baseline dMRI metrics and SARA scores (Supplementary Tables) were significant (type 1 error corrected) in SCA1 for the corpus callosum (*p* < 0.05 for MD, AD, and RD), right ICP (*p* = 0.044 in RD), bilateral SCP (*p* < 0.05 for MD and RD), and left sagittal stratum (*p* = 0.028 for FA). The association between baseline dMRI and ADL scores also showed significance in the corpus callosum (*p* < 0.05 for FA and RD) in SCA1 and right ICP (*p* = 0.002 for RD) in SCA2. The association between baseline dMRI and disease duration showed significance in the right corona radiata (*p* = 0.01 for RD) in SCA3.

Comparing the longitudinal changes (slopes) in dMRI metrics with changes in SARA showed significant association in the left posterior thalamic radiation (PTR-L, *p* < 0.03 for AD and MD) in SCA1 and the left SCP (*p* = 0.028 for RD) and left SLF (*p* = 0.046 for RD) in SCA3. No significant associations were detected between changes in dMRI metrics and ADL scores. The full statistics, including coefficients and corrected p-values for testing associations between clinical metrics and DTI metrics, were summarized in Supplementary Tables.

## Discussion

### Overview

In this study, we presented a multimodal evaluation of dMRI data in a cross-sectional approach and longitudinal changes in dMRI in a cohort of SCA1, 2, 3, and 6 gene carriers. This study identified differences in WM tracts and microstructure, comparing patients with SCAs with matched healthy controls. Although results were relatively consistent across analysis methods (ROI, TBSS, and FBA), certain differences were identified. This was particularly evident for FBA, which can model WM crossing fiber configurations from dMRI data, while ROI-based and TBSS analyses rely on the diffusion tensor model. Longitudinal ROI analysis also showed significant, albeit relatively small, within-subject changes in diffusion metrics. This is the first report of such finding in a SCA1 cohort using 3T dMRI. We find that our cross-sectional results are largely consistent with previous neuropathological and dMRI studies. This demonstrates the robustness of dMRI analysis in SCAs, and feasibility of dMRI to be used as a potential imaging biomarker.

### Cross-Sectional Analysis

ROI, TBSS, and FBA all consistently showed significant differences, between healthy controls and patients with SCAs, in several WM tracts. Lower FA along with higher diffusivity (MD, AD, and RD) in all SCAs than controls are consistent with WM microstructural alterations related to the disease. The MCP, along with both ICP and SCP, most consistently appeared as the regions that showed differences between patients and controls for SCA 1, 2, 3, and 6. Several WM tracts in the cerebrum, such as the cerebral peduncles or corticospinal tract and internal capsule, were also shown to have cross-sectional differences in both ROI and TBSS analyses for SCA1 and SCA2. SCA2 showed the most widespread alterations for all metrics in ROI, TBSS, and FBA analyses, followed by SCA1. This result is consistent with previously reported dMRI studies covering both SCA1 and SCA2 (20, 21, 25). SCA6 showed more limited alterations restricted to the cerebellum, as expected (4), and as identified by both FBA and TBSS with lower FA threshold of 0.1. Since prior volumetry analyses in SCA6 (43) have indicated alterations in the brainstem and pons, we expected similar changes in microstructural integrity in those regions. However, no significant differences were observed in either ROI, TBSS, or FBA results, indicating that the microstructural integrity may not have been affected despite volume loss in those regions. Note that the statistical power to detect such alterations might also be limited by our relatively small sample size.

We note that TBSS with a lower FA threshold (0.1) captures a larger number of WM tracts in peripheral regions, especially in the cerebellum. Although care needs to be taken when analyzing the cerebral white matter with this low FA threshold, the default value (0.2) eliminates numerous fine cerebellar tracts, which can be detrimental to the analysis of dMRI data for SCAs and other diseases with cerebellar involvement.

Comparison of different analysis methods (ROI, TBSS, and FBA) also shows how the results from various pipelines can be complementary. While ROI-based analysis enables the extraction of subject-specific metrics in their respective native space, TBSS analysis allows the identification of additional WM regions, which may not be available in templates and atlases used for automated ROI definitions. There also appears to be larger differences between FBA results and ROI-based or TBSS analyses. This is likely because both ROI-based and TBSS analyses are voxel-based and rely on DTI metrics such as FA, MD, AD, and RD, while FBA is tract-based and uses fiber crossing models. Note that we identified a loss in fiber density in the left corticospinal tract for SCA3 using FBA analysis, which was not detected in either ROI or TBSS analyses. Nonetheless, ROI-based analysis may be the most feasible candidate as a clinical imaging biomarker, since it does not require group-based spatial normalization as in TBSS or FBA pipelines. However, since the ROI analysis in this study utilized WM tracts defined in the JHU atlas (38), it was only able to identify cerebellar regions close to the brainstem and cerebellar peduncles. This could be a limiting factor for studying “pure cerebellar” disorders such as SCA6, which are characterized by widespread cerebellar alterations including the cortex. The use of a custom dMRI atlas with enhanced cerebellar definitions or improved segmentation of the cerebellum, similar to the T1-weighted template of cerebellum and corresponding atlas found in SUIT (44) and CERES (45), could mitigate this issue.

### Longitudinal Analysis

Longitudinal ROI-based analysis showed an increasing trend for MD in the MCP and right posterior thalamic radiation (PTR-R) in patients with SCA1. An increase in diffusivity is typically associated with axonal damage (46), and this trend therefore suggests progression of the disease. However, PTR-R did not show any significant differences in our cross-sectional analysis. Namely, even though MD significantly increased over time, the values found in patients were in the range of those found in healthy controls. Therefore, this effect may not be specifically associated with disease progression but could rather reflect aging-related changes. Another region with significant changes was the MCP, showing significant increases in AD. Unlike PTR-R, this region showed significant difference in our cross-sectional analysis, with clear separation in AD between patients and healthy controls. This suggests that AD in MCP could be a good biomarker for disease progression in SCA1. It is also interesting to note that increased AD in MCP was observed in the longitudinal dMRI analysis on Friedrich's Ataxia (47), suggesting the need for further studies in this particular region.

We also note the cases of three premanifest patients with SCA1 (Figure 6). The two premanifest patients with shorter predicted time to ataxia onset (4 years) at baseline showed AD values at MCP in the range of early stage patients with SCA1. One of these patients progressed to manifest stage in ~4 years, as predicted, while the other was already symptomatic ~1.5 years after baseline (Figure 6). The data from the first premanifest subject suggests that microstructural abnormalities may be detectable up to 4 years before ataxia onset. Because of the limited number of premanifest patients in our analysis, our results should be considered as preliminary. These findings also show the need for more comprehensive studies focusing on early and premanifest disease stages with greater number of patients.

### Association Between dMRI Metrics and Clinical Scores

Only diffusivity values showed significant associations with clinical scores. For SARA, only the SCA1 group (the largest group) showed an association with baseline dMRI metrics. We found that the SCP in SCA1 might be a good indicator of disease severity, as it demonstrated significant association with SARA as well as clear cross-sectional difference from healthy controls. Longitudinal changes in RD appeared to be significantly associated with changes in SARA in SCA1 for the left PTR (*p* < 0.03 for AD and MD) and in SCA3 for the left SCP and left SLF. In the case of SCA3, the result should be interpreted with caution, despite statistical significance, as there were only 4 longitudinal datasets. Nonetheless, this result could serve as a basis for future longitudinal studies with larger sample sizes. Note that no significant associations were found between longitudinal reductions in the volume of regions such as the cerebellum and brainstem measured at 1.5T and corresponding clinical scores (14), indicating that complimentary use of dMRI could be beneficial in detecting the longitudinal progression of SCAs.

### Limitations

Due to hardware limitations of the Trio platform, our HARDI acquisition used *b* = 1,500 s/mm^2^. However, MRtrix3 suggests dMRI data acquisition with higher *b*-values (*b*≥2,500 s/mm^2^), even though FBA is possible with lower *b*-values (42). Hence, future dMRI studies should address such technical considerations for accommodating FBA analysis.

The longitudinal datasets for SCA 2, 3, and 6 have a small sample size (*n* = 5/4/5, respectively), and therefore, many results did not reach statistical significance due to limited power. Moreover, we note that longitudinal data acquisitions could not always be performed at regular intervals. This was appropriately modeled in our statistical analysis but could also limit its power. Finally, the study was not initially designed to follow healthy control individuals longitudinally, but we note that such information would have been useful to model the effect of normal aging and take it into account in our longitudinal analysis.

## Conclusion

The evaluation of three different dMRI analysis pipelines in SCAs, which has not been reported previously, has demonstrated unique characteristics of each pipeline and benefits of pooling their results for a comprehensive understanding of the progression of microstructural abnormalities in SCAs. Our results show significant alterations of several WM tracts in SCA 1, 2, 3, and 6 compared to healthy volunteers. In addition, this first longitudinal dMRI analysis in SCA 1, 2, 3, and 6 at 3T suggests that AD in the MCP could be a good candidate imaging biomarker of disease progression for patients with SCA1. Preliminary results on premanifest patients show that microstructural abnormalities are detectable prior to ataxia onset and demonstrate the need for larger studies at the earliest stages of neurodegenerative changes.

## Data Availability Statement

The datasets generated for this study are available on request to the corresponding author.

## Ethics Statement

The studies involving human participants were reviewed and approved by University of Minnesota Institutional Review Board. The patients/participants provided their written informed consent to participate in this study.

## Author Contributions

CL, GÖ, and KB conceived and planned the experiments. JJ and DH carried out the data acquisition. YP and CL performed image analysis. YP, BG, and LE performed statistical analysis. YP, BG, IA, LE, GÖ, and CL contributed to the interpretation of the results. YP wrote the manuscript. All authors provided critical feedback and helped shape the research, analysis, and manuscript.

## Conflict of Interest

The authors declare that the research was conducted in the absence of any commercial or financial relationships that could be construed as a potential conflict of interest.
