# Corrigendum: Assessment of cerebral and cerebellar white matter microstructure in spinocerebellar ataxias 1, 2, 3, and 6 using diffusion MRI

**DOI:** 10.3389/fneur.2022.1038298

**Published:** 2022-09-29

**Authors:** Young Woo Park, James M. Joers, Bin Guo, Diane Hutter, Khalaf Bushara, Isaac M. Adanyeguh, Lynn E. Eberly, Gülin Öz, Christophe Lenglet

**Affiliations:** ^1^Department of Radiology, Center for Magnetic Resonance Research, University of Minnesota Medical School, Minneapolis, MN, United States; ^2^Division of Biostatistics, School of Public Health, University of Minnesota, Minneapolis, MN, United States; ^3^Department of Neurology, University of Minnesota Medical School, Minneapolis, MN, United States

**Keywords:** SCA1, SCA2, SCA3, SCA6, diffusion MRI, *Spinocerebeflar ataxias*

In the published article there was an error in the reference list as published. The reference list was submitted in the incorrect order. The revised reference list appears below.

The authors apologize for the above-mentioned errors and state that it does not affect the conclusions of the article in any way. The original version of this article has been updated.

## Publisher's note

All claims expressed in this article are solely those of the authors and do not necessarily represent those of their affiliated organizations, or those of the publisher, the editors and the reviewers. Any product that may be evaluated in this article, or claim that may be made by its manufacturer, is not guaranteed or endorsed by the publisher.
