# Supplementary figures and images for: Assessment of Cerebral and Cerebellar White Matter Microstructure in Spinocerebellar Ataxias 1, 2, 3, and 6 Using Diffusion MRI
Source: Front Neurol. 2020 Jun 4;11:411. doi: 10.3389/fneur.2020.00411 (PMC7287151; doi:10.3389/fneur.2020.00411)

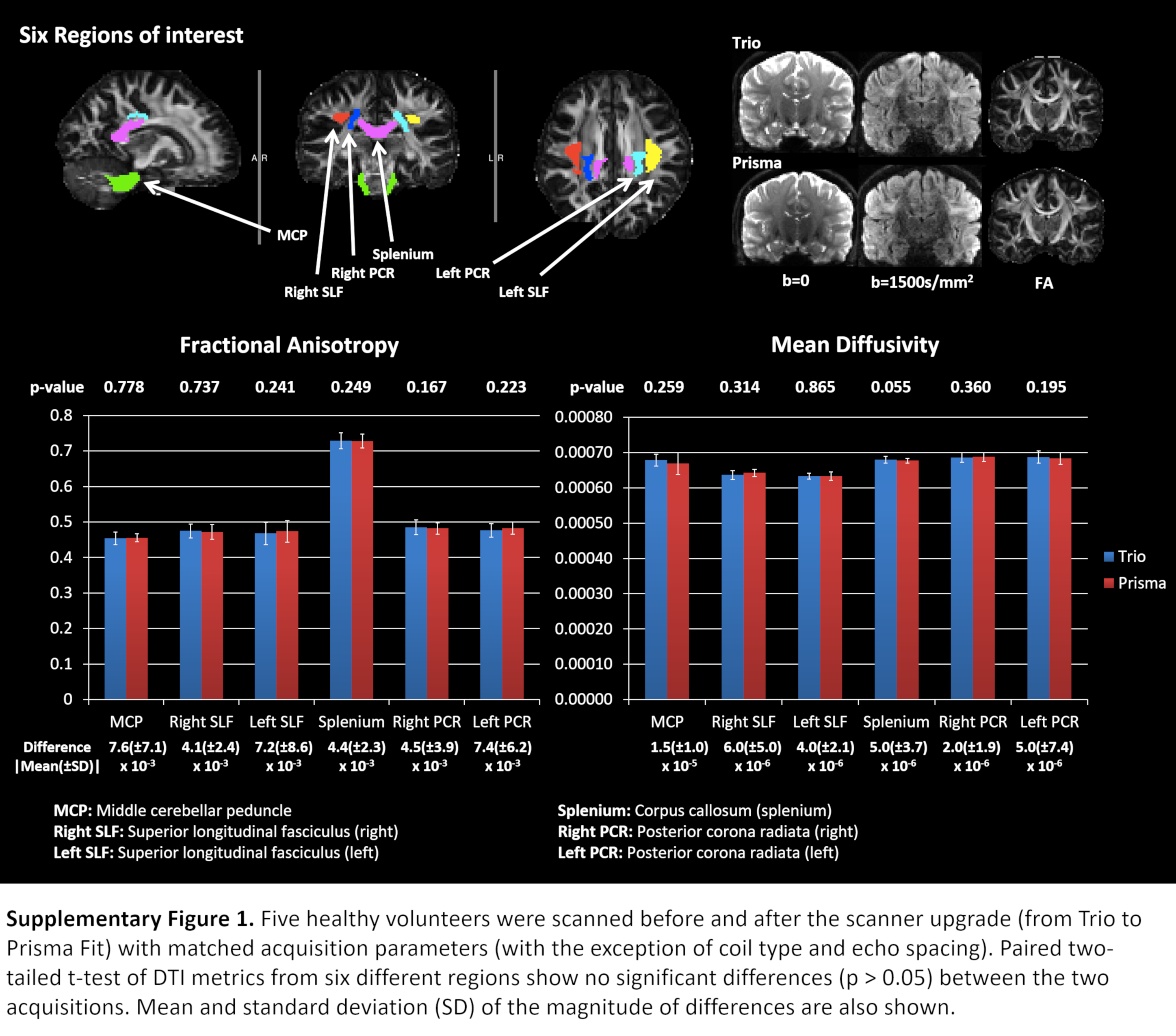

Supplement: Supplementary file 1 [file Image_1.JPEG]
